# Screening for optimal protease producing *Bacillus licheniformis* strains with polymer-based controlled-release fed-batch microtiter plates

**DOI:** 10.1186/s12934-021-01541-2

**Published:** 2021-02-23

**Authors:** Tobias Habicher, Tobias Klein, Jacqueline Becker, Andreas Daub, Jochen Büchs

**Affiliations:** 1 RWTH Aachen University, AVT – Biochemical Engineering, Forckenbeckstraße 51, 52074 Aachen, Germany; 2 BASF SE, Carl-Bosch-Straße 38, 67056 Ludwigshafen am Rhein, Germany

**Keywords:** Screening, Fed-batch, Microtiter plate, Protease, High-throughput

## Abstract

**Background:**

Substrate-limited fed-batch conditions have the favorable effect of preventing overflow metabolism, catabolite repression, oxygen limitation or inhibition caused by elevated substrate or osmotic concentrations. Due to these favorable effects, fed-batch mode is predominantly used in industrial production processes. In contrast, screening processes are usually performed in microtiter plates operated in batch mode. This leads to a different physiological state of the production organism in early screening and can misguide the selection of potential production strains. To close the gap between screening and production conditions, new techniques to enable fed-batch mode in microtiter plates have been described. One of these systems is the ready-to-use and disposable polymer-based controlled-release fed-batch microtiter plate (fed-batch MTP). In this work, the fed-batch MTP was applied to establish a glucose-limited fed-batch screening procedure for industrially relevant protease producing *Bacillus licheniformis* strains.

**Results:**

To achieve equal initial growth conditions for different clones with the fed-batch MTP, a two-step batch preculture procedure was developed. Based on this preculture procedure, the standard deviation of the protease activity of glucose-limited fed-batch main culture cultivations in the fed-batch MTP was ± 10%. The determination of the number of replicates revealed that a minimum of 6 parallel cultivations were necessary to identify clones with a statistically significant increased or decreased protease activity. The developed glucose-limited fed-batch screening procedure was applied to 13 industrially-relevant clones from two *B. licheniformis* strain lineages. It was found that 12 out of 13 clones (92%) were classified similarly as in a lab-scale fed-batch fermenter process operated under glucose-limited conditions. When the microtiter plate screening process was performed in batch mode, only 5 out of 13 clones (38%) were classified similarly as in the lab-scale fed-batch fermenter process.

**Conclusion:**

The glucose-limited fed-batch screening process outperformed the usual batch screening process in terms of the predictability of the clone performance under glucose-limited fed-batch fermenter conditions. These results highlight that the implementation of glucose-limited fed-batch conditions already in microtiter plate scale is crucial to increase the precision of identifying improved protease producing *B. licheniformis* strains. Hence, the fed-batch MTP represents an efficient high-throughput screening tool that aims at closing the gap between screening and production conditions.

## Background

Proteases are enzymes that catalyze the cleavage of proteins into smaller polypeptides or single amino acids. This ability offers a broad range of applications in industrial processes and products [1]. Thus, proteases are highly relevant in the technical enzyme market, especially in detergents [2]. Species from the genus *Bacillus* belong to the main producers of proteases. *Bacillus* species have high specific growth rates and the natural ability of secreting large amounts of proteins into the extracellular medium [3]. Nowadays, the production capabilities of protease producing *Bacillus* species are constantly improved by applying modern genetic engineering and molecular biology techniques [4–6]. With such techniques, large clone libraries are generated. Therefore, extensive screening campaigns have to be applied in order to identify strains with improved performance.

Screening of large clone libraries makes the use of small-scale high-throughput cultivation systems inevitable. The most frequently used small-scale cultivation systems for high-throughput screening applications are shaken bioreactors, such as microtiter plates [7, 8]. Microtiter plates are cost-efficient and have a simple and functional design, which enables a high degree of parallelization and automation [9, 10]. Basic correlations for mass transfer and power input further improved the understanding of microtiter plates [7]. However, microtiter plates were initially designed to be operated in batch mode. In contrast, the predominant mode in industrial protease production processes is fed-batch mode [2]. It is well known that with fed-batch mode, batch-related effects, such as overflow metabolism, osmotic inhibition and substrate inhibition, can be prevented [11]. Further, fed-batch mode allows to prevent catabolite repression. The prevention of catabolite repression plays an essential role when using promoters of genes related to catabolism. Catabolite controlled promoters are considered to be strong, and thus, commonly used with *Bacillus* species in industrial applications [4, 6, 12]. The induction and repression of these promoters, however, is directly coupled to the availability of the preferred carbon source, which most often is glucose. Thus, high glucose concentrations, as they occur in batch mode, have a repressive effect on protease formation [13].

It was demonstrated by transcriptome and proteome analyses that under batch and fed-batch conditions *B. licheniformis* cells are subject to completely different physiological conditions [14, 15]. The importance of avoiding such different physiological conditions during screening and production was already indicated by Tännler et al. [16]. Tännler et al. established a microtiter plate-based screening process for riboflavin producing *Bacillus subtilis* mutants with raffinose as carbon source. Raffinose mimicked, to some extent, carbon-limitation in fed-batch cultures, which enabled a more efficient identification of riboflavin overproducing mutants in comparison to batch conditions with glucose in excess. Scheidle et al. implemented a microtiter plate-based glucose release system to screen *Hansenula polymorpha* clones under glucose-limited fed-batch conditions [17]. The comparison of batch and fed-batch clone ranking revealed that clones were ranked completely differently in each mode. When comparing batch to glucose-limited fed-batch screening conditions with *Pichia pastoris*, similar results were found [18, 19]. Thus, the likelihood to identify improved strains for fed-batch fermentation processes is increased if the screening process is performed in fed-batch mode as well.

To establish similar physiological conditions during screening and production, microtiter plate-based cultivation systems enabling fed-batch operation have been developed [8, 20, 21]. The BioLector^®^ Pro (m2p-labs GmbH, Baesweiler, Germany) applies a microfluidic chip on a 48-well microtiter plate to continuously transfer a substrate solution from a reservoir well into a culture well, enabling 32 parallel cultivations [22, 23]. The micro-Matrix (Applikon Biotechnology, Delft, Netherlands) has built-in micro-injection valves that continuously add a substrate solution to wells of a 24-well microtiter plate. Besides high investment and operational costs for the BioLector^®^ Pro and the micro-Matrix, the possibility of feeding is coupled to the peripheral equipment of these devices. Thus, fed-batch cultivations in parallel microtiter plates are not feasible, which results in limited throughput or high investment costs.

**Fig. 1:**
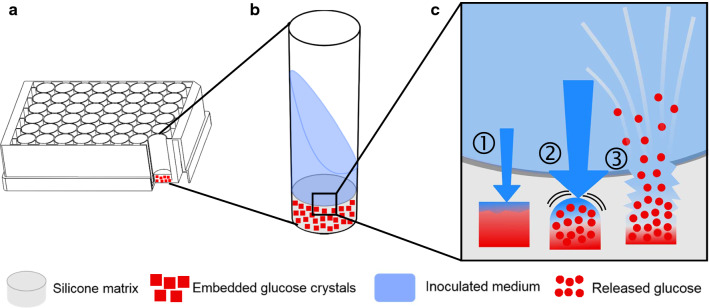
Principle of the polymer-based controlled-release fed-batch microtiter plate (fed-batch MTP). **a** Fed-batch MTP with cross section view into a single well. **b** Close-up illustration of a single round-well showing the silicone matrix with embedded glucose crystals on its bottom. **c** Working principle of the polymer-based controlled-release system adapted from Keil et al. [26]. ① Water diffuses into the silicone matrix and starts dissolving glucose crystals, thereby creating a highly concentrated glucose solution. ② Due to the osmotic pressure, more water diffuses into the cavity. ③ Little cracks and channels are established in the silicone matrix, which is followed by the release of the glucose solution into the culture medium

Microtiter plate-based fed-batch systems that are independent of peripheral equipment are enzyme-based and polymer-based glucose auto-delivery systems. The EnBase^®^ system (BioSilta, Oulu, Finnland) is based on the enzymatic degradation of starch, in which the glucose release rate can be adjusted via the enzyme concentration [24]. Thus, the EnBase® system is independent of scale and highly parallelizable, allowing high-throughput operation. However, the enzymatic system was found to be sensitive against changing cultivation conditions, such as pH and temperature [25]. Furthermore, many organisms, including *Bacillus* species, secrete amylases and proteases, leading to uncontrollable glucose release kinetics. An alternative glucose auto-delivery system is the fed-batch MTP (Feed Plate^®^, Kuhner shaker GmbH, Herzogenrath, Germany). It has a solid silicone matrix with embedded glucose crystals immobilized on the bottom of each well (Fig. 1). It is ready-to-use, disposable and parallelizable and thus applicable for high-throughput operation. In contrast to the EnBase^®^ system, the fed-batch MTP was demonstrated to be less sensitive against changing cultivation conditions [26]. Furthermore, amylases and proteases do not impair glucose release. Nowadays, different fed-batch MTP formats, ranging from 24, 48 and 96 round- and square-wells, with different release characteristics are available.

In this study, the previously established fed-batch process for protease expression with *B. licheniformis* in fed-batch MTP’s [27] was applied to perform a high-throughput and glucose-limited fed-batch screening campaign. The screening campaign was performed with a full set of *B. licheniformis* clones from two strain lineages without pre-selection (13 in total). The clone classification achieved under glucose-limited fed-batch conditions was compared to the clone classification achieved under batch conditions. Finally, the clone classification was validated with a lab-scale fed-batch fermenter operated under glucose-limited conditions. The presented microtiter plate-based glucose-limited fed-batch screening process aims at showing the advantage of establishing similar physiological conditions in microtiter plate screening and controlled fermenter processes in order to correctly identify protease producing *B. licheniformis* clones with improved performance.

## Results

### Preculture development

**Fig. 2:**
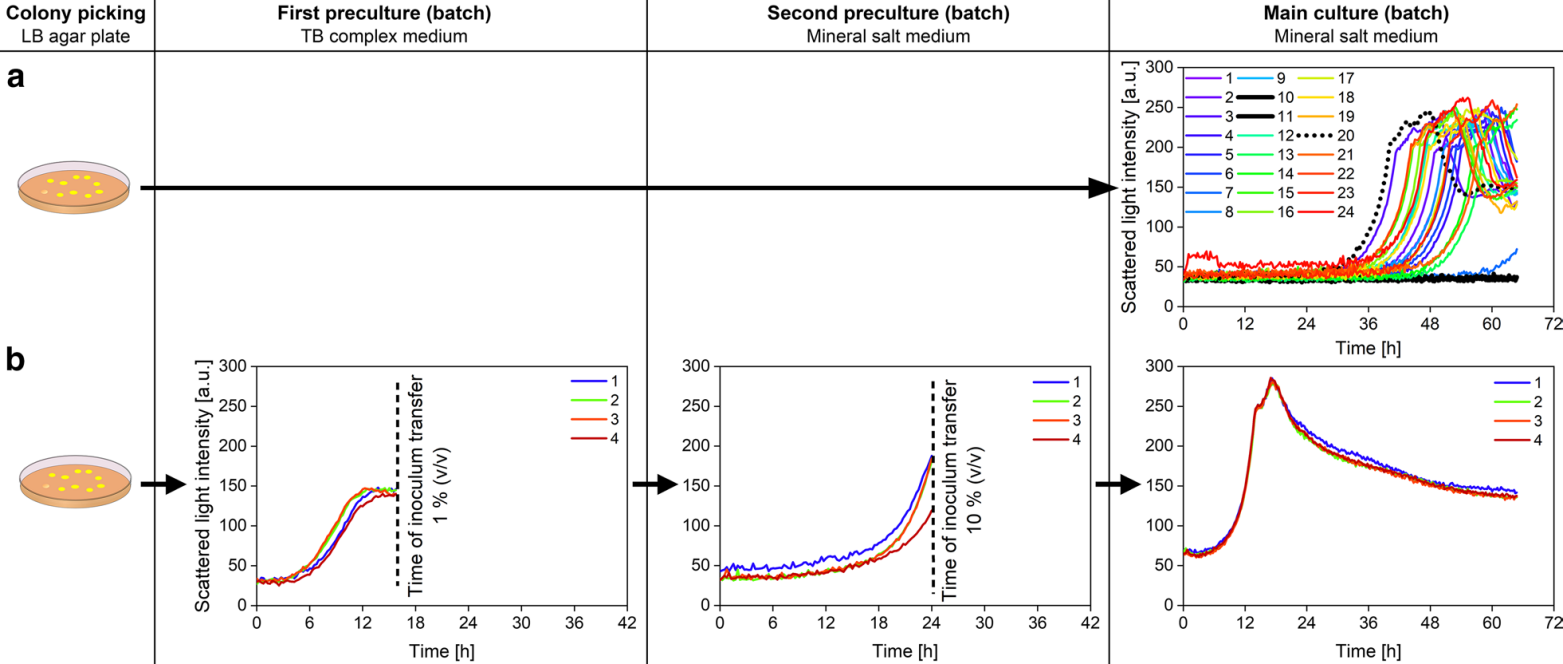
Preculture procedures for high-throughput screening processes with microtiter plates. Preculture procedures are exemplarily shown using one *B. licheniformis* strain. The arabic numbers stand for single colonies that were picked from agar plates and transferred into individual wells of a FlowerPlate^®^. The main culture represents a batch screening process. Scattered light intensities in the first, second and main culture represent biomass growth over time. **a** Direct inoculation of batch main culture. The fastest growing colony (colony 20) is indicated with a dashed black line. Colonies showing no growth after 65 h of cultivation (colony 10 and 11) are indicated with a solid black line. **b** Inoculation of batch main culture with a sequential preculture procedure. The first preculture was harvested after 16 h of cultivation as soon as each colony reached the scattered light plateau. 1% (v/v) of the filling volume, which corresponds to 8 µL, was transferred to the second preculture. The second preculture was harvested after 24 h of cultivation in the late exponential growth phase. 10% (v/v) of the filling volume, which corresponds to 80 µL, was transferred to the main culture. Cultivation conditions: FlowerPlate^®^, *n* = 1000 rpm, *d*_0_ = 3 mm, *V*_L, First preculture_ = 0.8 mL, *V*_L, Second preculture_ = 0.8 mL, *V*_L, Main culture_ = 0.88 mL, 30 °C

An often underestimated problem during screening processes is unequal growth of different clones in the main culture [28]. With unequal growth, individual clones are in uneven growth phases at the time of harvest, but are also exposed to the stationary phase for different lengths of time. This results in different clone rankings [29]. Variations in growth in the main culture are mainly caused by differences in the initial biomass concentration and in the duration of the lag-phase. Since such differences are attributed to the inoculum, different preculture procedures were investigated and are presented in Fig. 2.

In Fig. 2a, inoculation was realized by directly transferring single colonies from LB agar plates into individual wells of a batch main culture. The biomass growth (scattered light intensity) of the batch main culture was monitored online. The scattered light intensities of the batch main culture highlight unreproducible growth kinetics of individually picked colonies (Fig. 2a). The culture of colony 20, for example, has the shortest lag-phase and reaches the stationary phase after 65 h (Fig. 2a, dashed black line). In contrast, the cultures of colony 10 and 11 show no growth after 65 h (Fig. 2a, solid black lines).

In Fig. 2b, inoculation of a batch main culture was realized by performing the following sequential preculture procedure: 1. colony picking, 2. first preculture in complex medium, 3. second preculture in mineral medium. The first preculture was inoculated by picking single colonies from an agar plate. The scattered light intensities (colony 1–4) reveal comparable growth kinetics with a negligible lag-phase and a plateau in the scattered light intensity starting at around 12 h of cultivation. The inoculum transfer into the second preculture was conducted at 16 h.

The second preculture was inoculated with 1% (v/v) of the first preculture. The scattered light intensities (1–4) of the second preculture exhibit a similar growth behavior of all colonies with a short lag-phase of 6 h (Fig. 2b, second preculture). The second preculture was harvested in the late exponential growth phase at 24 h (Fig. 2b, second preculture).

**Fig. 3:**
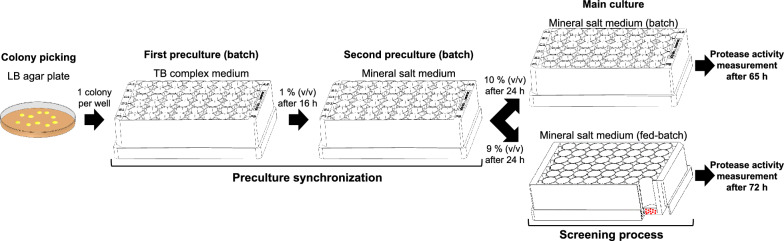
Flowchart of the applied screening procedure for high-throughput screening in batch and fed-batch mode. Precultures were synchronized applying a two-step preculture procedure. Synchronized precultures were used to inoculate batch and fed-batch main cultures. Batch main cultures were conducted in FlowerPlates^®^ and fed-batch main cultures in polymer-based controlled-release fed-batch microtiter plates (fed-batch MTP). The main cultures represent the screening processes in the respective mode. Cultivation conditions: FlowerPlate^®^ (batch), fed-batch MTP (fed-batch), *n* = 1000 rpm, *d*_0_ = 3 mm, *V*_L, First preculture_ = 0.8 mL, *V*_L, Second preculture_ = 0.8 mL, *V*_L, Main culture (batch)_ = 0.88 mL, *V*_L, Main culture (fed-batch)_ = 0.77 mL, 30 °C

The batch main culture was inoculated with 10% (v/v) of the second preculture. The scattered light intensities (1–4) of the batch main culture reveal synchronized growth of each colony with a negligible lag-phase (Fig. 2b, main culture). By means of the described preculture procedure, batch and fed-batch screening procedures were conducted as illustrated in Fig. 3.

### Determination of the number of replicates

**Fig. 4:**
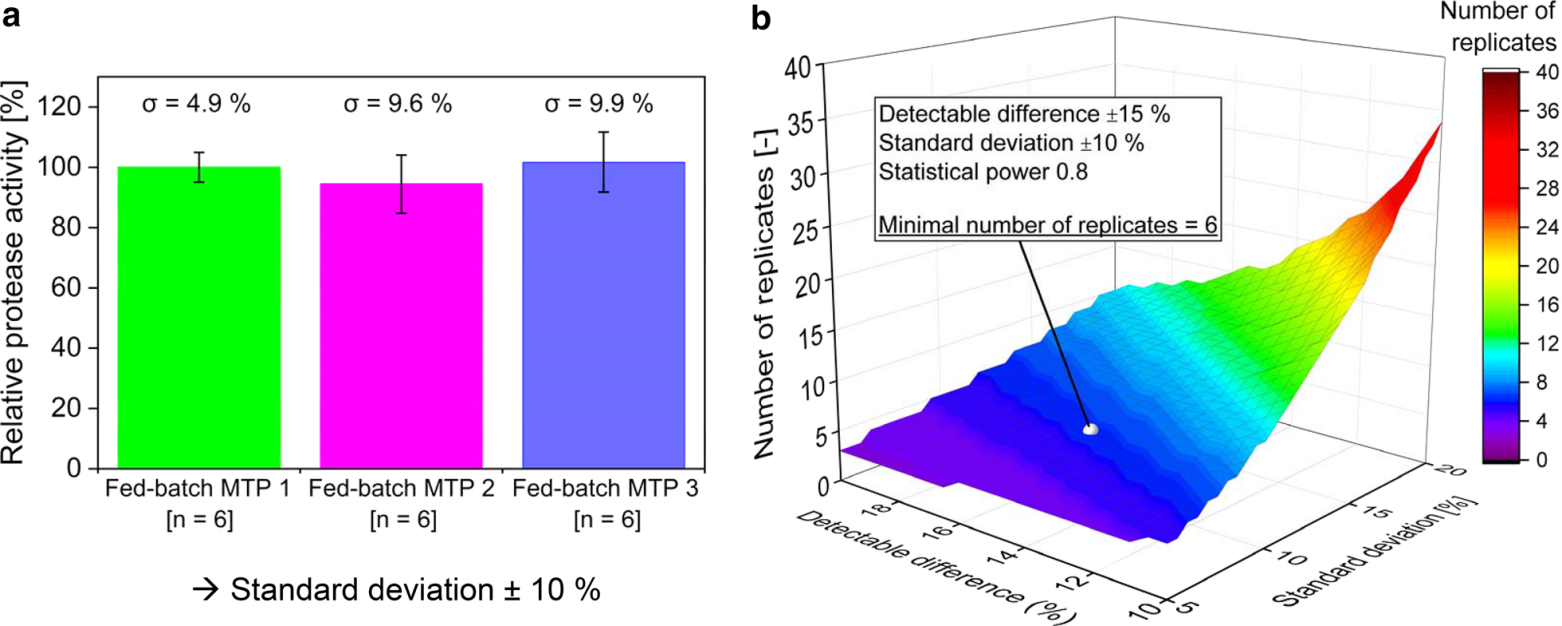
Statistical approach to determine the minimal number of replicates for a fed-batch screening campaign. **a** Mean relative protease activity of fed-batch cultivations with three individual fed-batch MTP’s (fed-batch MTP 1–3). The same *B. licheniformis* strain was used for all cultivations. The mean protease activity of fed-batch MTP 1 was normalized to 100%. Protease activities of fed-batch MTP 2 and 3 were set in relation to fed-batch MTP 1. Error bars symbolize the standard deviation (n = 6). An univariate ANOVA (*F* (2 15) = 1.21, *p* = 0.33) showed that there is no significant difference (*α* = 5%) between the investigated plates. Cultivation conditions: fed-batch MTP, *n* = 1000 rpm, *d*_0_ = 3 mm, *V*_L, Main culture (fed-batch)_ = 0.77 mL, 30 °C. **b** Number of replicates as a function of the detectable difference and standard deviation. The required detectable difference and statistical power are stated within the figure. The number of replicates was calculated with the MATLAB function *sampsizepwr* with a two-tailed *t*-test (normally distributed) as test type

The number of replicates needed to obtain statistically significant screening results has to be determined before conducting high-throughput screening experiments. Factors that influence the number of replicates are the standard deviation of the performance indicator after repetitions, the performance difference that has to be detected (detectable difference) and the demanded statistical power. With the protease activity as performance indicator, the required number of replicates for high-throughput screening experiments with fed-batch MTP’s used in this study was calculated. The results are depicted in Fig. 4.

In Fig. 4a, the final relative protease activities of glucose-limited fed-batch cultivations with fed-batch MTP’s are presented. Glucose-limited fed-batch cultivations were realized in six wells (n = 6) with three individual plates (fed-batch MTP 1–3) using the same *B. licheniformis* strain. Each plate was inoculated according to the procedure presented in Fig. 3. Relative protease activities of cultivations with fed-batch MTP’s (fed-batch MTP 1–3) show no significant difference on a significance level of 5% (Fig. 4a). The highest standard deviation of the relative protease activity within one plate is 9.9% (Fig. 4a, fed-batch MTP 3). Thus, the deviation of repetitive cultivations with fed-batch MTP’s is in the range of ± 10%.

In order to calculate the number of replicates, the value for the statistical power has to be selected. For the high-throughput screening in microtiter plates, a statistical power of 0.8 was chosen. Figure 4b shows the number of replicates as a function of the standard deviation and the detectable difference with a constant statistical power of 0.8. The number of replicates increases with an increasing standard deviation as well as with a decreasing detectable difference.

In order to calculate the minimal number of replicates, the value of the minimal detectable difference was chosen with ± 15%. With known minimal detectable difference of ± 15%, standard deviation of ± 10% and a statistical power of 0.8, the resulting minimal number of replicates is 6 (Fig. 4b). In other words, a minimum of 6 parallel glucose-limited fed-batch cultivations have to be conducted on a fed-batch MTP as used in this study to detect a clone having a 15% increased or decreased protease activity with a statistical power of 0.8.

### Comparison of batch and fed-batch screening

**Fig. 5:**
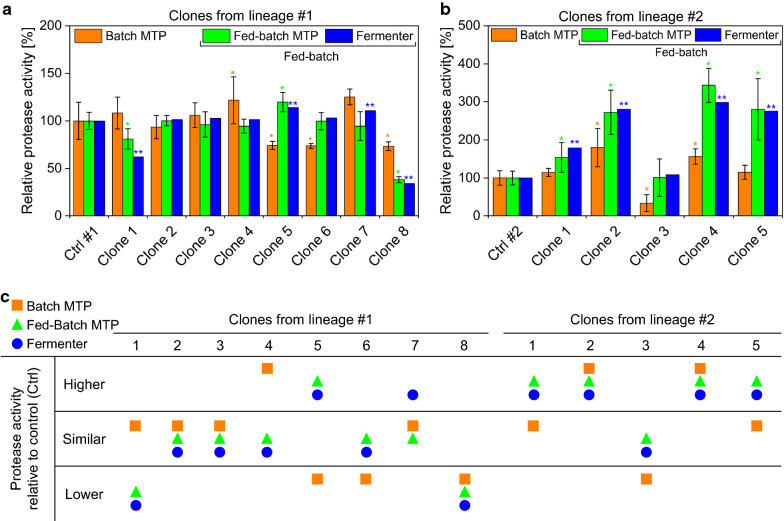
High-throughput screening of *B. licheniformis* clones in batch and fed-batch mode. **a** Mean relative protease activity of *B. licheniformis* clones of strain lineage #1 and **b** strain lineage #2 screened with microtiter plates (MTP) in batch (batch MTP) and fed-batch mode (fed-batch MTP) and validated with a lab-scale fed-batch fermenter under glucose-limited conditions (fermenter). The mean protease activities of the control strain of lineage one (Ctrl #1) and two (Ctrl #2) were normalized to 100%. Protease activities of clones were set in relation to the corresponding control strain cultivated under identical conditions. Error bars symbolize the standard deviation (batch MTP and fed-batch MTP, n = 6). Despite n ≥ 3, no error bars are shown for the lab-scale fed-batch fermenter (fermenter). Significant difference between each clone and control strain (two-tailed *t*-test with equal variance) is indicated with * for *p* < 0.1 and with ** for *p* < 0.05. **c** Performance classification of clones screened in batch (batch MTP) and fed-batch mode (fed-batch MTP) with validation under glucose-limited fed-batch conditions in a lab-scale fermenter (fermenter). The term “Similar” implies that there is no significant difference

*B. licheniformis* clones of two different lineages (#1 and #2) were screened under batch and glucose-limited fed-batch conditions according to the procedure presented in Fig. 3. Screening results were validated with a lab-scale fermenter operated under glucose-limited fed-batch conditions. The results of the microtiter plate (MTP)-based batch (batch MTP) and fed-batch screening (fed-batch MTP) as well as the experimental validation in the lab-scale fed-batch fermenter (fermenter) are summarized in Fig. 5.

Figure 5a and b depict the screening and fermenter results of the clone library of lineage #1 and #2, respectively. Based on these results, clones are classified into higher, lower or similar (no significant difference) performing clones relative to the control strain (Fig. 5c). The classification shown in Fig. 5c reveals that clones are classified differently with the batch MTP, the glucose-limited fed-batch MTP and the glucose-limited fed-batch process in a fermenter. The important question is, however, whether clones screened in batch mode (batch MTP) or fed-batch mode (fed-batch MTP) are classified similarly to the fed-batch fermenter process that mimics production conditions. Therefore, the clone classification of the batch screening (batch MTP) was compared to the classification achieved with the fed-batch fermenter process (fermenter). The comparison exhibits that the performance of 5 out of 13 clones is correctly predicted in batch screening mode, which corresponds to a predictability of 38%. The comparison of the clone classification achieved with the fed-batch screening (fed-batch MTP) and fed-batch fermenter process (fermenter) reveals that 12 out of 13 clones are classified similarly. This corresponds to a correct prediction of 92%.

## Discussion

### Preculture development

Assuming that the main culture shown in Fig. 2a represents a typical batch screening process that is terminated after 65 h, individual clones are harvested in completely different growth phases. One reason for unequal growth behavior in the main culture relates to the colony picking process. Colony picking causes a transfer of different colony volume and the picked colonies can be in unequal physiological stages [28]. These variations cannot be avoided by changing the picking method (by hand or by picking robot) or picking instrument (inoculation loop, toothpick, pipet tip, etc.) [29, 30]. However, although the colony picking method described in Fig. 2b was similar to the procedure in Fig. 2a, scattered light intensities (1–4) of the first preculture exhibit negligible lag-phases. The prevention of the lag-phases can be attributed to the applied culture medium. In Fig. 2a, colonies from complex LB agar plates were directly transferred into mineral salt medium of the main culture. The changing substrate availability from complex to mineral basis results in extended lag-phases and unpredictable growth kinetics. Besides the substrate source, also physical changes within the culture environment, as for example the medium osmolality, influence growth of *B. licheniformis* [31, 32]. By applying the sequential preculture procedure (Fig. 2b), colonies from complex LB agar plates were transferred into complex TB medium of the first preculture. Hence, the exemplarily shown cultivations using TB medium reveal efficient growth within the first preculture.

The positive effect on growth is not the only reason for using complex TB medium within the first preculture. Apart from the complex compounds, TB medium is supplemented with glycerol. In TB medium, glycerol represents the primary carbon source for *B. licheniformis*. The depletion of glycerol is indicated by a plateau of the scattered light intensity at approximately 12 h of cultivation (Fig. 2b, first preculture). At this point, the complex compounds act as slowly accessible substrate sources, which prevent *B. licheniformis* from undergoing carbon starvation. This results in equal growth conditions with equal biomass concentrations for each individually picked colony, which is indicated by the scattered light plateau (colony 1–4) reached after 16 h of cultivation (Fig. 2b, first preculture, Additional file 1). If *B. licheniformis* cultures face carbon starvation, the scattered light intensity exhibits a declining trend instead of showing a plateau (Fig. 2b, main culture; Additional files 1 and 2). Under carbon starvation, *Bacillus* cells exhibit cell lysis [33, 34] and morphological changes [35], resulting in decreasing scattered light intensities [36].

The reason for using a relatively small inoculum volume of 1% (v/v) to inoculate the second preculture was to minimize the transfer of unmetabolized complex compounds (Fig. 2b, second preculture). The short lag-phase is most probably caused by transferring the culture from complex to mineral medium. Diluting the complex compounds is not the only reason for introducing a second preculture. The second preculture also serves to adapt the *B. licheniformis* culture to the mineral salt medium, which is also applied in the main culture. However, when working with mineral salt media in the preculture, the time of inoculum transfer determines the inoculum quality. Since the mineral salt medium contains 20 g/L glucose as only carbon source, glucose depletion results in carbon starvation. Besides cell lysis [33, 34] and morphological changes [35], carbon starvation causes a variety of metabolic changes in *Bacillus* cultures [15, 37, 38]. To prevent the culture from undergoing these changes, the second preculture was harvested in the late exponential growth phase at 24 h (Fig. 2b, second preculture), prior to glucose depletion.

The batch main culture was inoculated with 10% (v/v) of the second preculture to ensure a high initial biomass concentration (Fig. 2b, main culture). This is of particular importance when the screening process is conducted with the fed-batch MTP. Due to the release principle of this microtiter plate (Fig. 1), glucose is released from the beginning of the cultivation (Additional file 3) [26, 27]. At this point, the biomass concentration is too low to consume all of the released glucose. This results in an initial batch phase. Its length is dependent on the initial biomass concentration [27]. Low initial biomass concentrations might cause an extended batch phase with oxygen limitation and overflow metabolism.

A commonly applied preculture procedure consists of a single preculture instead of a two-step procedure (Additional file 1). This single preculture can be performed either in complex medium or mineral salt medium. As described above, directly transferring picked colonies into mineral salt medium revealed substantial lag-phases with unequal growth (Fig. 2a), which does not result in synchronized cultures. Precultures performed in complex medium showed negligible lag-phases and equal growth, thereby enabling growth synchronization (Fig. 2b, Additional file 1). However, when performing a single preculture in complex medium, complex compounds are transferred into the main culture. At the same time, a short lag-phase was observed when transferring the culture from the complex medium to the mineral salt medium (Fig. 2b). Both, the transfer of complex compounds and lag-phases become crucial when working with fed-batch MTP’s. As mentioned above, the fed-batch MTP’s require high initial cell concentrations. Thus, with a single preculture it is difficult to find a trade-off between reaching high initial cell densities and reducing the amount of transferred complex compounds into the main culture. By introducing a second preculture with mineral salt medium, the complex compounds transferred to the main culture can be reduced, on the one hand, and the *B. licheniformis* culture can adapt to the mineral salt medium, on the other, which enables equal growth with negligible lag-phases in the main culture (Fig. 2b).

Unequal growth conditions can also be prevented with the use of a robotic platform (RoboLector®) consisting of a BioLector® and a liquid-handling robot [9, 39]. Applying fed-batch mode to synchronize growth of precultures is another functional and well-described procedure [28, 29]. However, with the used *B. licheniformis* strain, two sequential preculture cultivations in batch mode represent an easy to implement and efficient solution to synchronize the precultures.

### Determination of the number of replicates

In every high-throughput screening there is a trade-off between the number of replicates used for each strain and the number of different strains investigated. While the reliability increases with more replicates, the throughput decreases at the same time. Determination of the minimal number of replicates needed for a statistically sound evaluation of strain performance can be used to find an optimum between statistical reliability and throughput. However, for calculating the minimal number of replicates the standard deviation, the minimal detectable difference and the statistical power is required.

The standard deviation of repetitive cultivations with fed-batch MTP’s is in the range of ± 10% (Fig. 4a). This standard deviation represents the sum of individual deviations occurring throughout the entire screening procedure. This includes the analytical procedure of the protease activity measurement. However, the largest deviation derives from the glucose-limited fed-batch cultivation with the fed-batch MTP. It has been found that glucose release from fed-batch MTP’s exhibits a mean coefficient of variation of 4.5% [26]. This deviation in glucose release directly influences the final protease activity.

The results in Fig. 4b show that besides the standard deviation, the detectable difference influences the number of replicates. The minimal detectable difference describes to which extend the protease activity must at least increase or decrease in order to be detected with the demanded statistical power. In contrast to the standard deviation, the minimal detectable difference is a parameter that cannot be determined experimentally. The value of the minimal detectable difference relies for example on previous experiences with strain improvement programs. Since the number of replicates decreases with increasing minimal detectable difference (Fig. 4b), a clone that has a change in protease activity >  ± 15% is still securely detected with 6 replicates (statistical power ≥ 0.8). If only 3 replicates would be used, as often found in literature, only strains with a productivity increase >  ± 30% compared to the control strain could be identified with a standard deviation of ± 10% and a statistical power of 0.8 (Additional file 4a). On the other hand, using only 3 replicates but selecting for strains with ± 15% improvement at ± 10% standard deviation would result in a statistical power of only 0.3 (Additional file 4b).

The statistical power describes the probability of correctly rejecting the null hypothesis, i.e. correctly identifying clones with different protease activities. Therefore, the statistical power is an important measure regarding the reliability of screening results. The higher the statistical power, the higher the number of replicates needed to correctly identify clones with a different protease activity (Additional file 4b). In order to find a trade-off between statistical reliability and number of replicates (Additional file 4b), a statistical power of 0.8 was chosen for this study.

### Comparison of batch and fed-batch screening

The comparison of the predictability of batch (38%) and fed-batch screening mode (92%) highlights that implementing fed-batch conditions is essential to predict the clone performance similarly to the fed-batch fermenter that mimics production conditions. The necessity of implementing fed-batch conditions during screening is exemplarily discussed based on the results achieved with clone 4 and 5 from lineage #1 and clone 1 and 5 from lineage #2 (Fig. 5c). Clone 4 from lineage #1 exhibits a significantly higher protease activity under batch screening conditions (Fig. 5c, batch MTP). With the assumption that the screening process is only conducted in batch mode, clone 4 would be selected as a promising strain candidate and would be further investigated under lab-scale fed-batch conditions. Under these conditions, however, clone 4 shows no significantly higher protease activity and would be rejected (Fig. 5c, fermenter). Under fed-batch screening conditions, in contrast, clone 4 is correctly identified as a strain with no significantly higher protease activity (Fig. 5c, fed-batch MTP). Consequently, with a screening process conducted in fed-batch mode, the strain would not have been considered further, thereby saving time and costs. The results achieved with clone 5 from lineage #1 and clone 1 and 5 from lineage #2 represent an opposite scenario (Fig. 5c). Due to the similar (non-significant) or significantly lower performance achieved under batch screening conditions (Fig. 5c, batch MTP), these clones would directly be rejected. However, under fed-batch screening conditions, these clones were correctly identified as clones with a significantly higher protease activity than the control strain (Fig. 5c, fed-batch MTP and fermenter). This scenario shows that optimal protease producing clones can remain undetected under batch screening conditions, due to unequal physiological conditions between screening and production processes.

## Conclusion

High-throughput screening is crucial in strain optimization programs. Usually, the screening processes are performed with microtiter plates in batch mode. However, batch conditions can cause unfavorable phenomena that can be prevented by applying fed-batch conditions. Therefore, production processes are usually operated under glucose-limited fed-batch conditions. The fed-batch MTP used in this study and commercialized by Kuhner Shaker GmbH as Feed Plate^®^ is capable of establishing such glucose-limited fed-batch conditions in microtiter plates by continuously releasing glucose into the medium.

It was demonstrated that direct inoculation with colonies picked from agar plates resulted in unequal growth within the main culture. Thus, a sequential preculture procedure starting with colony picking was developed. By applying a two-step preculture procedure, equal growth conditions were achieved within the main culture. Based on this preculture procedure, the standard deviation of repetitive experiments with the fed-batch MTP was investigated. It was found that the standard deviation of the final protease activity of repetitive glucose-limited fed-batch cultivations is in the range of ± 10%. With known standard deviation of ± 10%, chosen minimal detectable difference of ± 15% and statistical power of 0.8, the minimal number of replicates was 6. Without calculating the number of replicates, there is the risk of randomly choosing a too low number of replicates. This could lead to a situation where a statistically sound identification of improved clones is not achievable. Thus, initially attaining some key numbers and adjusting the screening setup accordingly can drastically improve the success of a screening campaign.

The comparison of the batch and fed-batch screening underlines the necessity of implementing glucose-limited fed-batch conditions already during early strain screening campaigns. In batch mode, 38% of the investigated *B. licheniformis* clones were classified similarly to the lab-scale fed-batch fermenter operated under glucose-limited conditions. In contrast, when applying glucose-limited fed-batch conditions in the microtiter plate, 92% of the investigated clones were classified similarly to the lab-scale fed-batch fermenter process. These results demonstrate that the performance of a potential production strain can only be evaluated if the physiology of the strain is comparable between screening and production conditions. For a production process relying on glucose-limited fed batch, the fed-batch MTP’s used in this study proved to be capable of implementing such conditions. Hence, the fed-batch MTP represents an efficient high-throughput fed-batch screening tool that aims at closing the gap between screening and production.

## Methods

### Strains

Protease producing *Bacillus licheniformis* clones were provided by BASF SE (Ludwigshafen am Rhein, Germany). A heterologous protease was produced belonging to the group of subtilisin proteases originating from *Bacillus lentus* [40]. Screening was performed with a full set of clones of two different *B. licheniformis* strain lineages, both expressing the same protease. No pre-selection was made regarding the choice of clones. The clone library of strain lineage one (#1) consisted of eight clones (clone 1–8) and one control strain, which is referred to as Ctrl #1. The clone library of strain lineage two (#2) consisted of five clones (clone 1–5) and one control strain, which is referred to as Ctrl #2. Both lineages derive from rational strain engineering and the fed-batch MTP system was used to evaluate the impact of these strain modifications in comparison to the respective parent strain.

### Media

The chemicals applied for media preparation were of analytical grade and purchased from Sigma-Aldrich Chemie GmbH (Steinheim, Germany), if not stated otherwise.

Agar plates were used for colony picking and were based on complex lysogeny broth (LB) medium. The LB medium contained per liter: 10 g tryptone (Pancreatic digest from casein, Sigma-Aldrich Chemie GmbH, Steinheim, Germany), 10 g NaCl and 5 g yeast extract (Biospringer, Maisons-Alfort, France). The pH was adjusted to 7.0 with 5 M NaOH. Subsequently, 15 g of bacteriological agar were dissolved in 1 L of LB medium and sterilized by autoclaving (121 °C for 20 min). At a temperature of approximately 50 °C the LB agar was poured into plates.

The complex terrific broth (TB) medium was used for the first preculture and contained per liter: 10 g glycerol (C_3_H_8_O_3_), 12 g tryptone (Pancreatic digest from casein, Sigma-Aldrich Chemie GmbH, Steinheim, Germany), 24 g yeast extract (Biospringer, Maisons-Alfort, France), 12.54 g K_2_HPO_4_ and 2.31 g KH_2_PO_4_. After autoclaving (121 °C for 20 min), the medium was stored at 4 °C for not longer than 6 months.

A mineral salt medium was used for the second preculture and for main cultures in microtiter plates. The mineral salts and trace elements contained in the mineral salt medium are described in WO/2020/169,564 [41]. The concentration of the respective chemicals was adapted to the lower amount of glucose that was used for batch and fed-batch cultivations compared to the > 200 g/L of glucose added in the above mentioned patent application. To maintain pH in a reasonable range the medium contained 0.2 M MOPS buffer and the initial pH was adjusted to 7.5. For batch and fed-batch cultivations in microtiter plates, the medium was supplemented with 20 and 0 g/L glucose, respectively. Glucose release in fed-batch MTP’s is shown in Additional file 3. Except for the glucose concentration, the mineral salt medium formulation was identical for batch and fed-batch cultivations with microtiter plates.

### Preculture

The preculture procedure consisted of sequential steps and was started by plating glycerol cryo stocks on LB agar plates, followed by an overnight incubation at 30 °C. Single colonies were picked with toothpicks from the LB agar plate and used to inoculate the first preculture. Each colony was then transferred into one well of a FlowerPlate^®^ (MTP-B48-B, m2p labs GmbH, Baesweiler, Germany) filled with *V*_L, First preculture_ = 0.8 mL of complex TB medium. After 16 h, 1% (v/v) of the culture broth of the first preculture (8 µL) was used to inoculate the second preculture. The second preculture was performed in a FlowerPlate^®^ with a filling volume *V*_L, Second preculture_ = 0.8 mL using a mineral salt medium. After 24 h, 10% and 9% (v/v) of the culture broth of the second preculture were used to inoculate the batch and fed-batch main culture, respectively. Cultivations with FlowerPlates^®^ were carried out on a BioLector^®^ device (m2p labs GmbH, Baesweiler, Germany). The BioLector^®^ device enabled online biomass monitoring via scattered light intensity at a wavelength *λ* = 620 nm (gain = 20) (Fig. 2, Additional files 1 and 2). To reduce evaporation and prevent cross contamination, FlowerPlates^®^ were sealed with a gas-permeable sealing foil (F-GPR48-10, m2p labs GmbH, Baesweiler, Germany). Cultivations were performed with a shaking frequency *n* = 1000 rpm, a shaking diameter *d*_0_ = 3 mm, at 30 °C and a relative humidity *φ* > 75%.

### Main culture

Main cultures in microtiter plates were performed in batch and fed-batch mode using a mineral salt medium. Batch cultivations were performed in FlowerPlates^®^ on a BioLector^®^ device (m2p labs GmbH, Baesweiler, Germany) with a filling volume *V*_L, Main culture (batch)_ = 0.88 mL. Fed-batch cultivations were performed in a 48-round-well fed-batch MTP’s (Feed Plate^®^, Art. Nr.: SMFP08004, Kuhner Shaker GmbH, Herzogenrath, Germany) on an orbital climo shaker (Multitron Pro, Infors AG, Bottmingen, Switzerland) with a filling volume *V*_L, Main culture (fed-batch)_ = 0.77 mL. To reduce evaporation and prevent cross contamination, both FlowerPlates^®^ and fed-batch MTP’s were sealed with a gas-permeable sealing foil (F-GPR48-10, m2p labs GmbH, Baesweiler, Germany). Cultivations were performed with a shaking frequency *n* = 1000 rpm, a shaking diameter *d*_0_ = 3 mm, at 30 °C and a relative humidity *φ* > 75%. The cultivation conditions in microtiter plate-based batch and fed-batch main cultures were adjusted so that an oxygen limitation was prevented (Additional file 2) [27]. Culture acidification was prevented by using MOPS buffer, thereby keeping the pH in the neutral range between 7 and 7.5 (Additional file 2) [27]. Batch and fed-batch cultivations were terminated after 65 and 72 h, respectively. The harvested culture broth was filtered (0.45 µm filter) and the protease activity analyzed. Figure 3 gives an overview of the applied screening procedure.

The fed-batch fermenter process was conducted in a lab-scale stirred tank reactor system (DASGIP^®^ Bioblock 1 L fermentation system, Eppendorf AG, Jülich, Germany). The system was operated with a glucose feeding strategy resulting in glucose-limited fed-batch conditions. pH was controlled above 7 by addition of ammonia solution (NH_4_OH). The dissolved oxygen tension (DOT) was controlled at a setpoint of 30%. The fermentation process has been described in detail in WO/2020/169,564 [41].

### Protease assay

Protease activity measurement was based on the method developed by DelMar et al. [42] and on the experimental procedure described by Meissner et al. [35]. The following information was previously published by Habicher et al. [13]. The assay was carried out in a microplate reader (Synergy 4, BioTek, Winooski, VT, USA) at a wavelength of 405 nm. The concentration of the substrate stock solution of N-succinyl-alanine-alanine-proline-phenylalanine-p-nitroanilide (N-Suc-AAPF-pNA, Bachem AG, Bubendorf, Switzerland) was 60 mg/mL using water free dimethyl sulfoxide (DMSO). Stocks were stored at -20 °C for not longer than 6 months. Prior to use, the substrate stock was diluted 1/50 with 0.1 M Tris HCl buffer, pH 8.6, 0.1% (w/v) Brij 35 (reaction buffer). The reaction was started by adding 100 µL of diluted substrate stock to 50 µL of sample in a 96-well microtiter plate with clear and flat bottom (Rotilabo microtest plates, N°9293.1, Carl Roth GmbH + Co. KG, Karlsruhe, Germany). The absorption measurement was conducted at 30 °C for 15 min. Samples were diluted with reaction buffer to keep the absorption at 405 nm between 0.05 and 1. Protease activity was calculated based on the change of absorption at 405 nm, the extinction coefficient ε of 8,900 1/M/cm and the pathlength of 0.43 cm. Protease activities were normalized to a reference process (Fig. 4) or to control strains (Ctrl #1 and #2, Fig. 5). Within this work, protease activities are referred to as relative protease activities.

## Supplementary Information

**Additional file 1.** Course of the scattered light intensity of a single preculture with complex TB medium and main culture with mineral salt medium. Cultures are exemplarily shown using one *B. licheniformis* strain. The Arabic numbers stand for single colonies that were picked from agar plates and transferred into individual wells of a FlowerPlate^®^. Scattered light intensity in the preculture and main culture shows biomass growth over time. In the batch preculture with complex TB medium scattered light intensities reach a plateau at around 12 h, which lasts for at least 18 h. Thus, the batch preculture was harvested after 16 h of cultivation. 1% (v/v) of the preculture filling volume, which corresponds to 8 µL, was transferred to the batch main culture with mineral salt medium. Cultivation conditions: FlowerPlate^®^, *n* = 1000 rpm, *d*_0_ = 3 mm, *V*_L_ = 0.8 mL, 30 °C.

**Additional file 2.** Batch main culture of *B. licheniformis* with mineral salt medium. The main culture was inoculated according to the procedure depicted in Fig. 3. Shadows symbolize the standard deviation of cultivations with four individually picked colonies (n = 4). Roman numbers divide the course of the cultivation into three distinct phases: **I** exponential growth phase on glucose, **II** growth phase on overflow metabolites, and **III** carbon starvation phase. Glucose depletion (dashed line between I and II) is visible in the scattered light intensity and indicated by the switch of the pH and dissolved oxygen tension (DOT) into an upward direction. The pH switch is caused by the consumption of previously accumulated overflow metabolites. Overflow metabolite consumption (II) is recognizable in the course of the scattered light intensity and the DOT. The scattered light intensity increases again whereas the DOT exhibits another downward spike. Carbon starvation (dashed line between II and III) is indicated by the DOT approaching 100 % and the constantly decreasing scattered light intensity. Cultivation conditions: FlowerPlate^®^ with pH and DOT optode, *n* = 1000 rpm, *d*_0_ = 3 mm, *V*_L__, Main culture (batch)_ = 0.88 mL, 30 °C.

**Additional file 3.** Glucose release over time using the fed-batch MTP. a Glucose release within an individual well (n = 6) of the fed-batch MTP with the mineral salt medium used within this study. Cultivation conditions: *n* = 1000 rpm, *d*_0_ = 3 mm, *V*_L_= 0.77 mL, 30 °C. **b** Glucose release within an individual well (n = 3) of the fed-batch MTP with V3 mineral medium. Adapted with permission from Habicher et al. (2020). In **a** and **b** similar fed-batch MTP’s (48-round-well) were used and show a comparable release of glucose. Keil et al. (2019) provided an equation to calculate glucose release under different media and cultivation conditions in 96-square-well fed-batch MTP’s.

**Additional file 4.** Detectable difference and statistical power as function of the number of replicates. **a** Detectable difference as function of the number of replicates. The statistical power and the standard deviation were set to 0.8 and ± 10 %, respectively. **b** Statistical power as function of the number of replicates. The detectable difference and the standard deviation were set to ± 15 % and ± 10 %, respectively. Calculations were done with the MATLAB function sampsizepwr with a two-tailed t-test (normally distributed) as test type.

## Data Availability

The data that support the findings of this study are available from BASF SE but restrictions apply to the availability of these data, which were used under license for the current study, and so are not publicly available. Data are however available from the authors upon reasonable request and with permission of BASF SE.
