# Supplementary material for: Screening for optimal protease producing Bacillus licheniformis strains with polymer-based controlled-release fed-batch microtiter plates
Source: Microb Cell Fact. 2021 Feb 23;20:51. doi: 10.1186/s12934-021-01541-2 (PMC7903736; doi:10.1186/s12934-021-01541-2)
Supplement: Supplementary file 1 — Additional file 1. Course of the scattered light intensity of a single preculture with complex TB medium and main culture with mineral salt medium. Cultures are exemplarily shown using one B. licheniformis strain. The Arabic numbers stand for single colonies that were picked from agar plates and transferred into individual wells of a FlowerPlate®. Scattered light intensity in the preculture and main culture shows biomass growth over time. In the batch preculture with complex TB medium scattered light intensities reach a plateau at around 12 h, which lasts for at least 18 h. Thus, the batch preculture was harvested after 16 h of cultivation. 1% (v/v) of the preculture filling volume, which corresponds to 8 µL, was transferred to the batch main culture with mineral salt medium. Cultivation conditions: FlowerPlate®, n = 1000 rpm, d0 = 3 mm, VL = 0.8 mL, 30 °C. [file 12934_2021_1541_MOESM1_ESM.docx]

**Additional files**


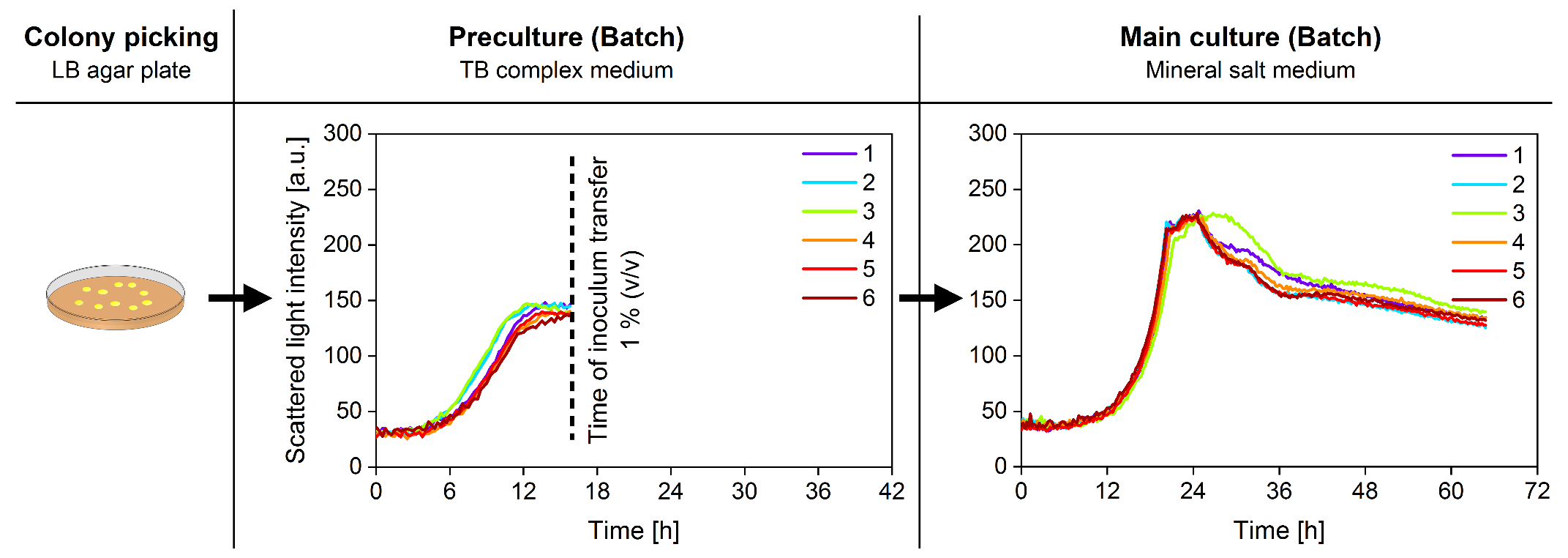


**Additional file 1.** Course of the scattered light intensity of a single preculture with complex TB medium and main culture with mineral salt medium. Cultures are exemplarily shown using one *B. licheniformis* strain. The Arabic numbers stand for single colonies that were picked from agar plates and transferred into individual wells of a FlowerPlate^®^. Scattered light intensity in the preculture and main culture shows biomass growth over time. In the batch preculture with complex TB medium scattered light intensities reach a plateau at around 12 h, which lasts for at least 18 h. Thus, the batch preculture was harvested after 16 h of cultivation. 1 % (v/v) of the preculture filling volume, which corresponds to 8 µL, was transferred to the batch main culture with mineral salt medium. Cultivation conditions: FlowerPlate^®^, *n* = 1000 rpm, *d_0_* = 3 mm, *V_L_* = 0.8 mL, 30 °C.
