# Supplementary material for: Screening for optimal protease producing Bacillus licheniformis strains with polymer-based controlled-release fed-batch microtiter plates
Source: Microb Cell Fact. 2021 Feb 23;20:51. doi: 10.1186/s12934-021-01541-2 (PMC7903736; doi:10.1186/s12934-021-01541-2)
Supplement: Supplementary file 2 — Additional file 2. Batch main culture of B. licheniformis with mineral salt medium. The main culture was inoculated according to the procedure depicted in Fig. 3. Shadows symbolize the standard deviation of cultivations with four individually picked colonies (n = 4). Roman numbers divide the course of the cultivation into three distinct phases: I exponential growth phase on glucose, II growth phase on overflow metabolites, and III carbon starvation phase. Glucose depletion (dashed line between I and II) is visible in the scattered light intensity and indicated by the switch of the pH and dissolved oxygen tension (DOT) into an upward direction. The pH switch is caused by the consumption of previously accumulated overflow metabolites. Overflow metabolite consumption (II) is recognizable in the course of the scattered light intensity and the DOT. The scattered light intensity increases again whereas the DOT exhibits another downward spike. Carbon starvation (dashed line between II and III) is indicated by the DOT approaching 100 % and the constantly decreasing scattered light intensity. Cultivation conditions: FlowerPlate® with pH and DOT optode, n = 1000 rpm, d0 = 3 mm, VL, Main culture (batch) = 0.88 mL, 30 °C. [file 12934_2021_1541_MOESM2_ESM.docx]

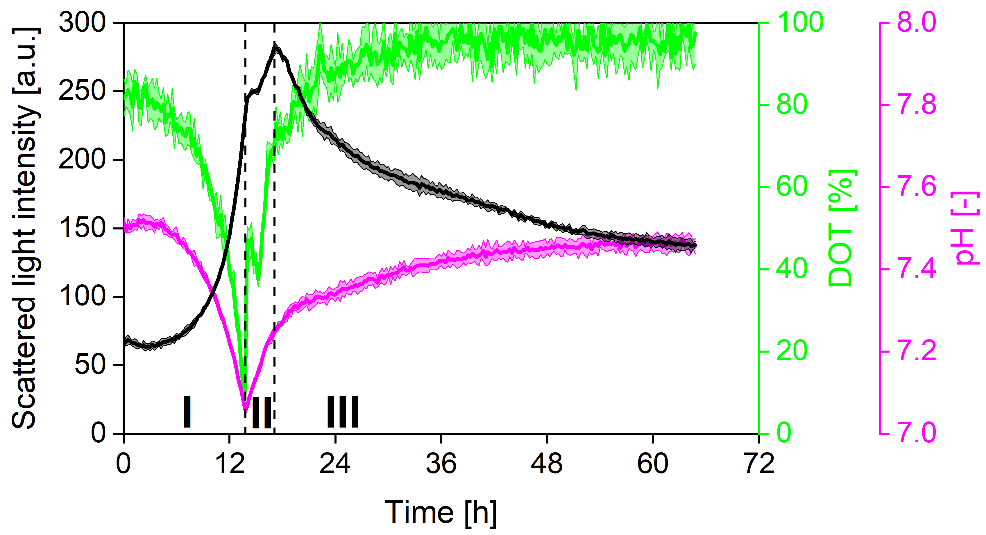


**Additional file 2.** Batch main culture of *B. licheniformis* with mineral salt medium. The main culture was inoculated according to the procedure depicted in Fig. 3. Shadows symbolize the standard deviation of cultivations with four individually picked colonies (n = 4). Roman numbers divide the course of the cultivation into three distinct phases: **I** exponential growth phase on glucose, **II** growth phase on overflow metabolites, and **III** carbon starvation phase. Glucose depletion (dashed line between I and II) is visible in the scattered light intensity and indicated by the switch of the pH and dissolved oxygen tension (DOT) into an upward direction. The pH switch is caused by the consumption of previously accumulated overflow metabolites. Overflow metabolite consumption (II) is recognizable in the course of the scattered light intensity and the DOT. The scattered light intensity increases again whereas the DOT exhibits another downward spike. Carbon starvation (dashed line between II and III) is indicated by the DOT approaching 100 % and the constantly decreasing scattered light intensity. Cultivation conditions: FlowerPlate^®^ with pH and DOT optode, *n* = 1000 rpm, *d*_0_ = 3 mm, *V*_L, Main culture (batch)_ = 0.88 mL, 30 °C.
