# Supplementary material for: Screening for optimal protease producing Bacillus licheniformis strains with polymer-based controlled-release fed-batch microtiter plates
Source: Microb Cell Fact. 2021 Feb 23;20:51. doi: 10.1186/s12934-021-01541-2 (PMC7903736; doi:10.1186/s12934-021-01541-2)
Supplement: Supplementary file 3 — Additional file 3. Glucose release over time using the fed-batch MTP. a Glucose release within an individual well (n = 6) of the fed-batch MTP with the mineral salt medium used within this study. Cultivation conditions: n = 1000 rpm, d0 = 3 mm, VL= 0.77 mL, 30 °C. b Glucose release within an individual well (n = 3) of the fed-batch MTP with V3 mineral medium. Adapted with permission from Habicher et al. (2020). In a and b similar fed-batch MTP’s (48-round-well) were used and show a comparable release of glucose. Keil et al. (2019) provided an equation to calculate glucose release under different media and cultivation conditions in 96-square-well fed-batch MTP’s. [file 12934_2021_1541_MOESM3_ESM.docx]

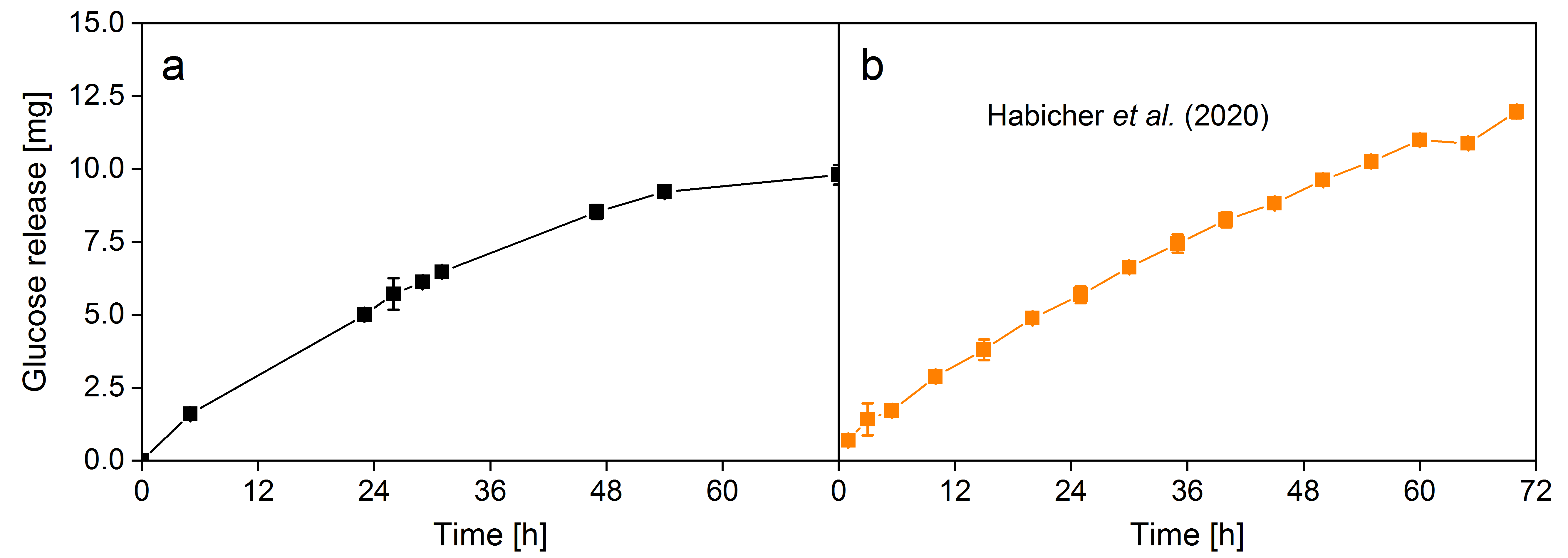


**Additional file 3.** Glucose release over time using the fed-batch MTP. **a** Glucose release within an individual well (n = 6) of the fed-batch MTP with the mineral salt medium used within this study. Cultivation conditions: *n* = 1000 rpm, *d*_0_ = 3 mm, *V*_L_= 0.77 mL, 30 °C. **b** Glucose release within an individual well (n = 3) of the fed-batch MTP with V3 mineral medium. Adapted with permission from Habicher *et al.* (2020). In **a** and **b** similar fed-batch MTP’s (48-round-well) were used and show a comparable release of glucose. Keil et al. (2019) provided an equation to calculate glucose release under different media and cultivation conditions in 96-square-well fed-batch MTP’s.
