# Supplementary material for: Screening for optimal protease producing Bacillus licheniformis strains with polymer-based controlled-release fed-batch microtiter plates
Source: Microb Cell Fact. 2021 Feb 23;20:51. doi: 10.1186/s12934-021-01541-2 (PMC7903736; doi:10.1186/s12934-021-01541-2)
Supplement: Supplementary file 4 — Additional file 4. Detectable difference and statistical power as function of the number of replicates. a Detectable difference as function of the number of replicates. The statistical power and the standard deviation were set to 0.8 and ± 10 %, respectively. b Statistical power as function of the number of replicates. The detectable difference and the standard deviation were set to ± 15 % and ± 10 %, respectively. Calculations were done with the MATLAB function sampsizepwr with a two-tailed t-test (normally distributed) as test type. [file 12934_2021_1541_MOESM4_ESM.docx]

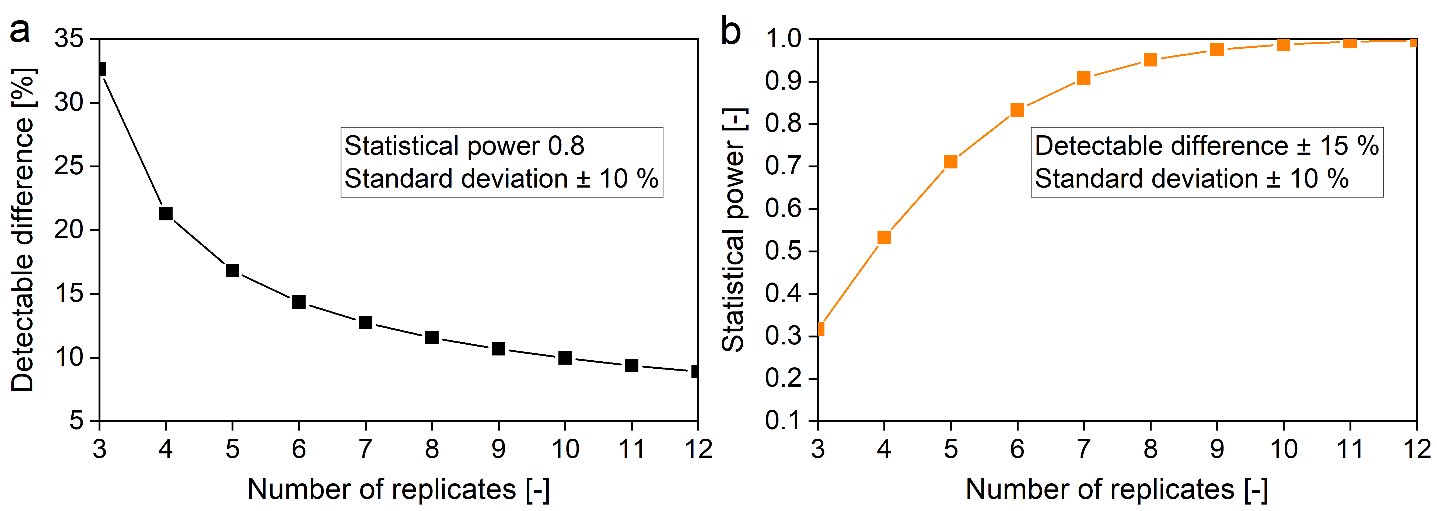


**Additional file 4.** Detectable difference and statistical power as function of the number of replicates. **a** Detectable difference as function of the number of replicates. The statistical power and the standard deviation were set to 0.8 and ± 10 %, respectively. **b** Statistical power as function of the number of replicates. The detectable difference and the standard deviation were set to ± 15 % and ± 10 %, respectively. Calculations were done with the MATLAB function *sampsizepwr* with a two-tailed *t*-test (normally distributed) as test type.
